# Continuous negative‐pressure wound therapy improves the survival rate of skin grafts and shortens the time required for skin graft survival

**DOI:** 10.1111/srt.13865

**Published:** 2024-07-19

**Authors:** Masato Tsuchiya, Toshihiro Kushibiki, Toshifumi Yamashiro, Yoshine Mayumi, Miya Ishihara, Ryuichi Azuma

**Affiliations:** ^1^ Department of Plastic and Reconstructive Surgery National Defense Medical College Tokorozawa Saitama Japan; ^2^ Department of Medical Engineering National Defense Medical College Tokorozawa Saitama Japan

**Keywords:** cytokine, growth factor negative‐pressure incubator, topical negative pressure, vacuum‐assisted closure, vascularity

## Abstract

**Background:**

The effectiveness of negative‐pressure wound therapy (NPWT) in skin graft fixation has been demonstrated in several clinical studies. However, in vitro and in vivo studies on skin graft fixation with NPWT have been scarce. In this in vivo study, we aimed to determine whether NPWT fixation enhances skin graft survival and how it contributes to improving skin graft survival biologically.

**Materials and methods:**

We harvested skin from the bilateral abdominal wall of 88 mice after anesthetizing them. Full‐thickness skin grafts (FTSGs) were performed on contralateral harvest sites, and grafts were fixed using NPWT (continuous and intermittent modes), conventional compression methods, and wrapping with polyurethane foam as a control group. On days 5 and 10 of grafting, the survival rates of the FTSGs were evaluated. Immunohistopathological analysis and measurement of the expression levels of vascular endothelial growth factor (VEGF), basic fibroblast growth factor (FGF‐2), and epidermal growth factor (EGF) were performed.

**Results:**

The survival rates of FTSG in the continuous NPWT group were significantly higher than those in the other groups. The number of capillaries in the dermis was significantly higher in the continuous NPWT group than in the other groups. In the wound bed, VEGF levels were significantly higher in both NPWT groups than in the other groups.

**Conclusion:**

Continuous NPWT increases the survival rate of FTSGs and shortens the duration of skin graft survival.

## 1 INTRODUCTION

Soft tissue coverage of skin defect wounds is a common problem in patients sustaining burns, ulcers, traumatic injuries, and wounds after the excision of a malignant tumor. Although several maneuvers for covering skin defects have been reported, skin grafting is one of the most effective and least invasive techniques.^1^ However, it is often associated with partial or complete necrosis of the skin grafts, which prolongs hospital stays and increases hospital expenses.^2^ Moreover, a long immobilization period is required to take skin grafts, which delays rehabilitation and leads to a lower quality of life.^3^, ^4^ Hence, many efforts have been made to improve skin graft survival and shorten immobilization periods; these include hyperbaric oxygen therapy, administration of various growth factors, and the minced micrograft technique.^5^, ^6^, ^7^, ^8^, ^9^ However, these therapies are currently in the investigation stage.

Negative‐pressure wound therapy (NPWT) is a new fixation technique for improving skin graft survival.^10^, ^11^, ^12^, ^13^, ^14^, ^15^, ^16^, ^17^, ^18^, ^19^, ^20^, ^21^ In this method, polyurethane foam with an open‐cell structure is placed on the wound surface and a suction pressure is applied using a vacuum source. Morykwas and Argenta were the first to report the efficacy of NPWT in promoting wound healing.^22^ They reported that NPWT increased blood flow levels, rates of formation of the granulation tissue, and survival rates of the random‐pattern flap and decreased the bacterial count on the wound in pig models. Subsequently, many in vitro and in vivo studies have shown that NPWT promotes wound healing through the following actions: wound contraction caused by negative pressure,^23^, ^24^ generation of various growth factors and cytokines from the wound bed caused by mechanical stress,^25^, ^26^, ^27^ improvement of bacterial clearance for wound irrigation, and elimination of excessive exudate.^22^, ^28^ The effectiveness of NPWT in skin graft fixation has been demonstrated in several clinical studies.^29^, ^30^ However, there have been no in vitro or in vivo studies on skin graft fixation using NPWT. Thus, the biological mechanisms through which NPWT fixation contributes to improving the survival rates of skin grafts remain unclear.

In this in vivo study, we aimed to determine whether NPWT fixation enhances skin graft survival and investigated the mechanism through which it contributes to skin graft survival. Moreover, we evaluated the most suitable NPWT method for skin graft survival.

## 2 MATERIALS AND METHODS

### 2.1 Mouse full‐thickness skin graft (FTSG) model

All animal experiments in this study were performed in accordance with the ARRIVE guidelines, and the experimental protocol was approved by the Defense Medical College Animal Experiment Ethics Committee (approval number: 190057). All procedures were performed in accordance with the relevant guidelines and regulations of the National Defense Medical College.

**FIGURE 1:**
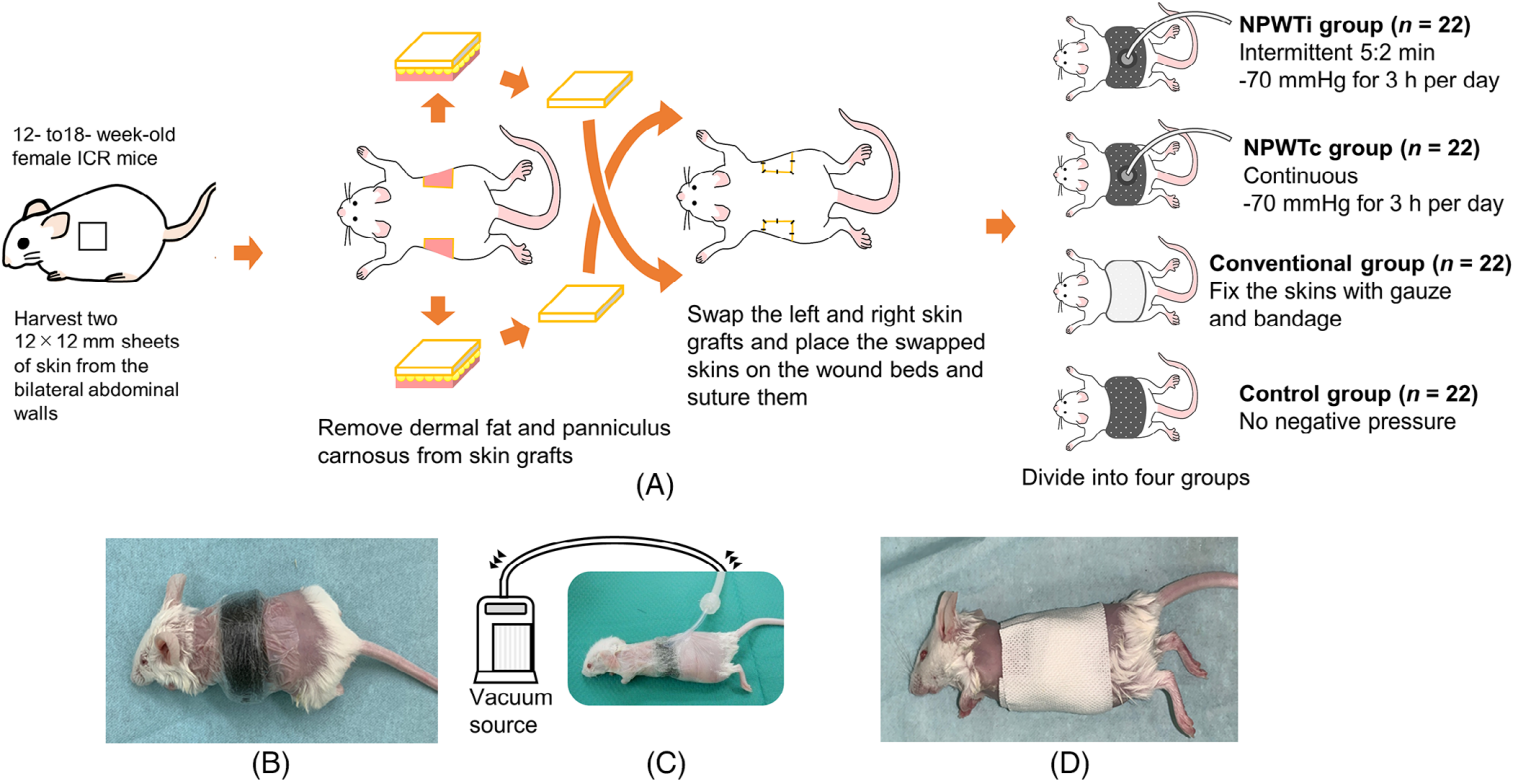
Procedure for making the skin graft model and assigning mice to each group on day 1. (A) An outline of the procedure on day 1. (B) NPWT model. (C) NPWT model attached to the vacuum source with power on. (D) Conventional model. ICR, Institute of Cancer Research; NPWTc, Continuous negative‐pressure wound therapy; NPWTi, Intermittent negative‐pressure wound therapy.

Twelve to eighteen‐week‐old female Institute of Cancer Research mice (Japan SLC, Shizuoka, Japan) were used for all experiments. Mice were maintained on standard laboratory diets and water ad libitum. On day 1, mice were anesthetized using an intraperitoneal injection of 0.3 mg/kg medetomidine, 4 mg/kg midazolam, and 5 mg/kg butorphanol tartrate. The skin hair on the whole body was shaved and depilated 1 day before the subsequent procedure. Two sheets of 12 mm square full‐thickness skin were harvested from the bilateral abdominal walls using a knife. Dermal fat and panniculus carnosus of the harvested skin were removed. Each skin piece was grafted onto the contralateral skin defect and fixed with eight sutures at the corners and middle of each edge (Figure 1).

### 2.2 Skin graft fixation using NPWT and conventional compression methods

**FIGURE 2:**
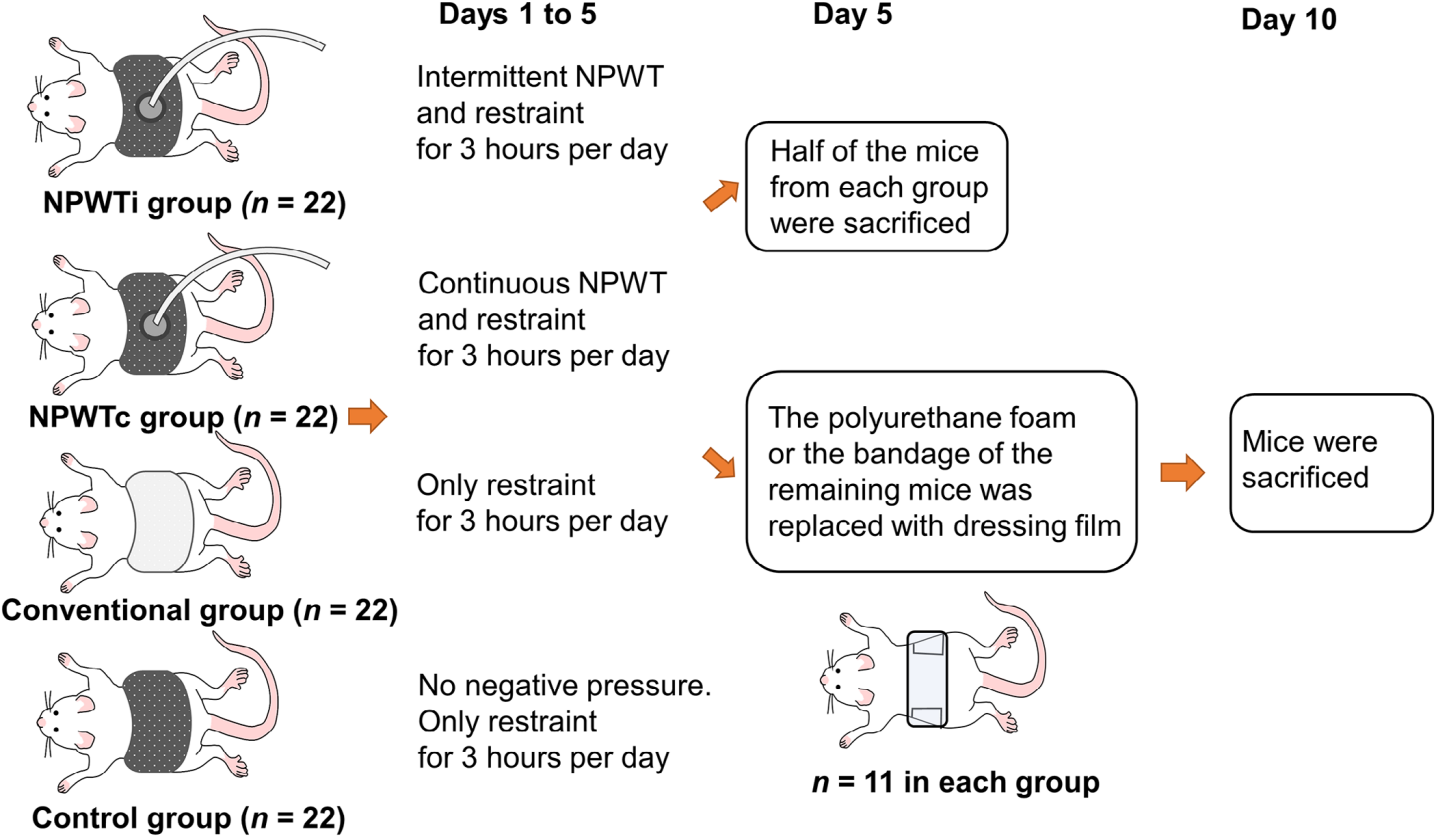
Experimental schedule. Mice in the NPWTi group were subjected to intermittent negative pressure cycling from ‐70 mmHg for 5 min to 0 mmHg for 2 min for 3 h per day. Mice in the NPWTc group were subjected to a continuous negative pressure of ‐70 mmHg for 3 h per day. Mice in the conventional group were wrapped in an adhesive bandage with folded gauze over the graft after skin grafting. Mice in the control group were wrapped with foam and dressing films and were not subjected to negative pressure. After each treatment on days 1 to 5, 11 randomly selected mice from each group were euthanized via cervical dislocation for further examination. For the remaining mice in each group, the black polyurethane foam or adhesive bandage with folded gauze was removed on day 5, and the mice were placed on the adhesive dressing film. On the 10th day, the mice were euthanized using cervical dislocation for further examination. NPWTc, Continuous negative‐pressure wound therapy; NPWTi, Intermittent negative‐pressure wound therapy.

Sixty‐six mice were assigned to the NPWT treatment group. The FTSGs were covered with black polyurethane foam with an open‐cell structure (RENASYS foam filler kit, Smith and Nephew, Watford, UK) and adhesive dressing films (Airwall, Kyowa, Osaka, Japan). The dorsal skin between the bilateral FTSG sites was also bridged using the same material. A pressure‐resident tube (EX tube, Nipro, Osaka, Japan) was placed as a drainage tube on top of the foam, embedded in the adhesive dressing film, and connected to a vacuum source (HAMA SERVO‐DRAIN 3000, Innomedics, Tokyo, Japan) (Figures 1 and 2).

The mice assigned to the NPWT group were divided into three subgroups. Mice in group 1 were subjected to intermittent negative pressure cycling from ‐70 mmHg for 5 min to 0 mmHg for 2 min for 3 h per day in a Ballman cage (Natsume Seisakusho, Tokyo, Japan) on days 1−5 (NPWTi group). Mice in group 2 were subjected to a continuous negative pressure of ‐70 mmHg for 3 h per day in a Ballman cage on days 1−5 (NPWTc group). Mice in group 3 were attached to foam and dressing films and were not subjected to negative pressure (control group). However, they were restrained in a Ballman cage for 3 h per day on days 1−5. After negative‐pressure treatment on day 5, 11 randomly selected mice from each group were euthanized using cervical dislocation for further examination.

Twenty‐two mice were assigned to the conventional group. Mice were wrapped in an adhesive bandage with folded gauze over the grafts after grafting, as described previously.^31^ They were restrained in a Ballman cage for 3 h per day on day 1−5. On day 5, 11 randomly selected mice from this group were sacrificed using cervical dislocation for further examination.

For the remaining mice in each group, the black polyurethane foam or adhesive bandage was removed on day 5 and the mice were placed on adhesive dressing film. On day 10, the mice were euthanized using cervical dislocation for further examination (Figures 1 and 2).

### 2.3 Assessment of skin graft survival and the criteria for selecting specimens for subsequent experiments

Digital photographs of the graft were taken on days 5 and 10 to analyze survival rates. The viability of each graft was assessed based on its appearance, color, and texture, as described previously.^9^ The number of pixels corresponding to the surface area of the surviving graft was calculated using the ImageJ software (National Institutes of Health, MD, USA). Survival rates were calculated as the average survival rates of bilateral skin grafts.

After determining the survival rates, the skin graft and wound bed with one of the highest survival rates were used for subsequent experiments. When the survival rates for both sides of the grafts were less than 50%, the specimens were excluded. Harvested skin grafts were used for immunohistochemical examinations and measuring the levels of growth factors.

### 2.4 Immunohistochemical examination

Skin grafts were placed in 10% formalin for 24 h and subsequently embedded in paraffin. The sections were processed routinely. They were subjected to immunohistochemical examination using CD31 antibodies (Abcam, Cambridge, UK) to count the number of capillary vessels in the dermis of the grafts. For staining, antigen retrieval was performed on paraffin‐embedded sections using 10 mM citrate buffer at 125°C for 10 min. Endogenous peroxidase was inactivated using 3% H_2_O_2_ and nonspecific binding of the antibodies was blocked using Blocking One (Nacalai Tesque, Kyoto, Japan). The sections were then incubated with primary and secondary antibodies. They were treated with 3, 3′‐diaminobenzidine‐4HCl, and Mayer's hematoxylin nuclear counterstain, according to the standard protocol.

Vascular profiles were characterized by positive staining for CD31 in structures with an identifiable vascular lumen, and the number of capillaries per square millimeter was calculated using the ImageJ software.

### 2.5 Quantitation of growth factors in the wound bed

The levels of growth factors expressed in each wound bed were measured. RIPA lysis and extraction buffer (500 µL) and Halt protease inhibitor cocktail (5 µL; Thermo Fisher Scientific, MA, USA) were added to each wound bed. The wound beds were cut into small fragments using scissors and incubated at 4°C for 30 min. The specimens were homogenized using a Physcotron homogenizer (Microtec, Chiba, Japan) at 30 000 rpm for 1 min. The samples were incubated again at 4°C for 30 min and centrifuged at 10 000 rpm for 1 min three times after homogenization. The supernatant, thus obtained, was used for the measurement of vascular endothelial growth factor (VEGF), basic fibroblast growth factor (FGF‐2), and epidermal growth factor (EGF) levels using a Mouse Quantikine ELISA Kit (R&D Systems, MN, USA) according to the manufacturer's instructions. A small amount of the supernatant was used to measure protein concentration with a Pierce BCA Protein Assay Kit (Thermo Fisher Scientific, MA, USA). All protein concentrations were normalized to the protein content of the cell layer using the BCA Protein Assay Kit. The data were analyzed using the JMP Pro 15.2.0 software (SAS Institute Japan Inc., Tokyo, Japan).

### 2.6 Statistical analysis

Data are expressed as mean ± standard error of the mean (SEM). Statistical analysis of the differences in survival rates of FTSGs between days 5 and 10 was performed using the Student's *t*‐test. Other experiments were analyzed using the Tukey–Kramer honestly significant difference test. All statistical analyses were performed using the JMP Pro 15.2.0 software. Statistical significance was set at *p* < 0.05.

## 3 RESULTS

### 3.1 Gross observations

All mice could tolerate the creation of full‐thickness skin wounds and grafts without complications. Specimens from the three mice assigned to the control group were excluded from further examination because their survival rates were < 50% on day 5. On day 10, the number of mice with survival rates < 50% was three in the control group, two in the conventional group, and one each in the NPWTc and NPWTi groups.

The survival rates of FTSGs on day 5 were significantly lower in the control group (67.9 ± 8.89%) than in the NPWTc (92.8 ± 1.15%, *p *= 0.0038), NPWTi (90.4 ± 1.35%, *p *= 0.0099), and conventional (87.5 ± 3.10%, *p *= 0.030) groups. On day 10, the survival rates were significantly higher in the NPWTc group (86.7 ± 5.97%) than in the NPWTi (57.9 ± 6.72%, *p *= 0.0272), control (53.9 ± 6.14 %, *p* = 0.0095), and conventional (53.4 ± 8.64 %, *p* = 0.0081) groups.

**FIGURE 3:**
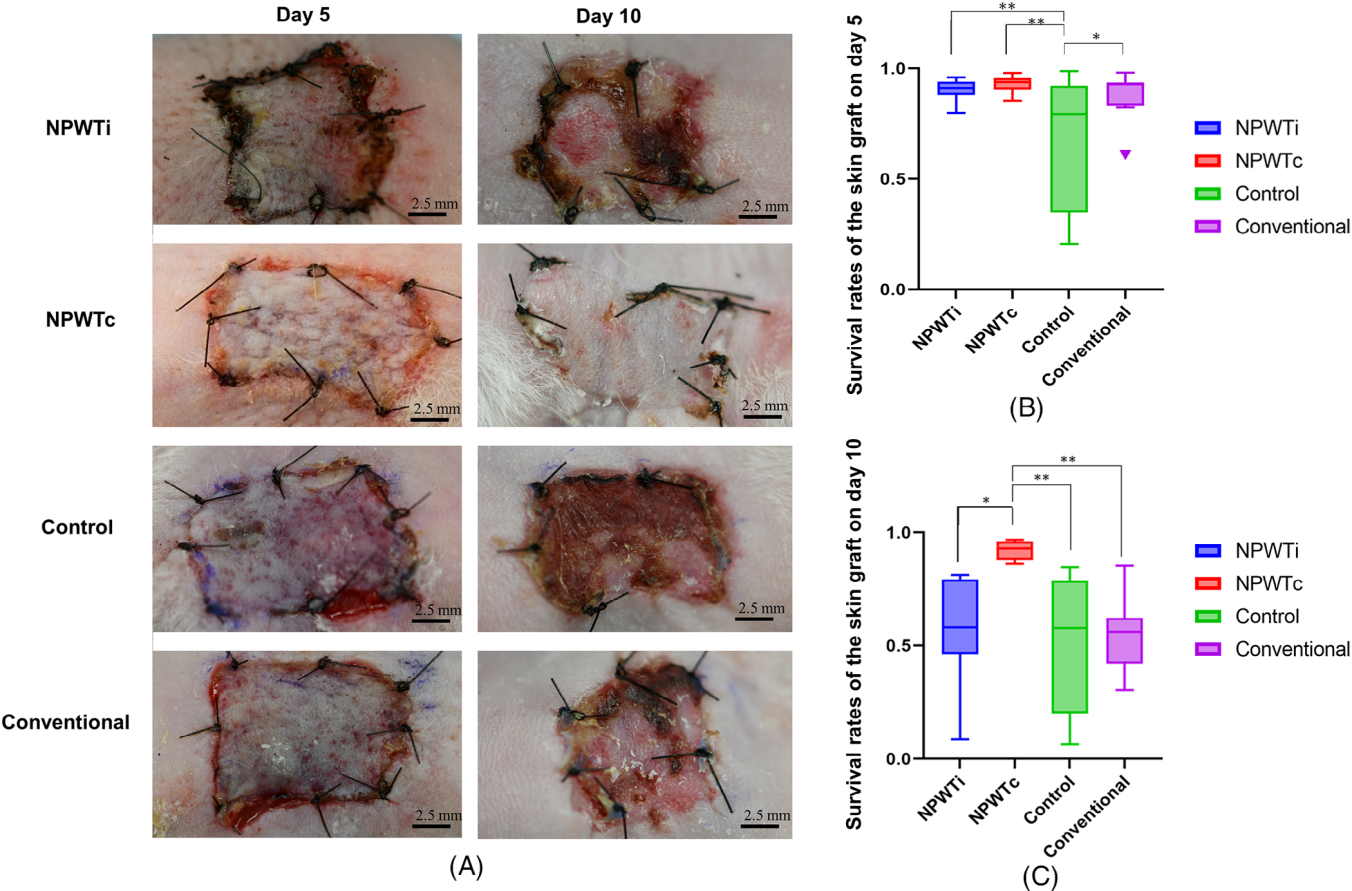
Survival rates of the skin grafts. (A) Representative examples of the skin grafts in each group. (B) Comparison of survival rates of skin grafts on day 5. On day 5, the survival rates of the skin grafts were significantly lower in the control group than in the NPWTc, NPWTi, and conventional groups. *n* = 11 in each group. (C) Comparison of the survival rates of skin grafts on day 10. On day 10, the survival rates of the skin grafts were significantly higher in the NPWTc group than in the NPWTi, control, and conventional groups. *n* = 11 in each group. * *p* < 0.05. ** *p* < 0.01. NPWTc, Continuous negative‐pressure wound therapy; NPWTi, Intermittent negative‐pressure wound therapy. Scale bar = 2.5 mm.

The survival rates in the NPWTi (*p *< 0.0001) and conventional (*p *< 0.0001) groups on day 10 were lower than those on day 5 (Figure 3 and Table 1).

**TABLE 1:** Survival rates of the skin grafts in each group on days 5 and 10.

	Survival rates of the skin grafts (%)		
Group	Day 5	Day 10	*p*‐value
NPWTi	90.4 ± 1.35	57.9 ± 6.72	<0.0001
NPWTc	92.8 ± 1.15	86.7 ± 5.97	0.3345
Control	67.9 ± 8.89	53.9 ± 6.14	0.2729
Conventional	87.5 ± 3.10	53.4 ± 8.64	<0.0001

*Note*: *n *= 11 in each group. The survival rates of skin grafts are expressed as mean ± SEM.

Abbreviations: NPWTc, Continuous negative‐pressure wound therapy; NPWTi, Intermittent negative‐pressure wound therapy.

### 3.2 Histological evaluation of vascularization at FTSG sites

**FIGURE 4:**
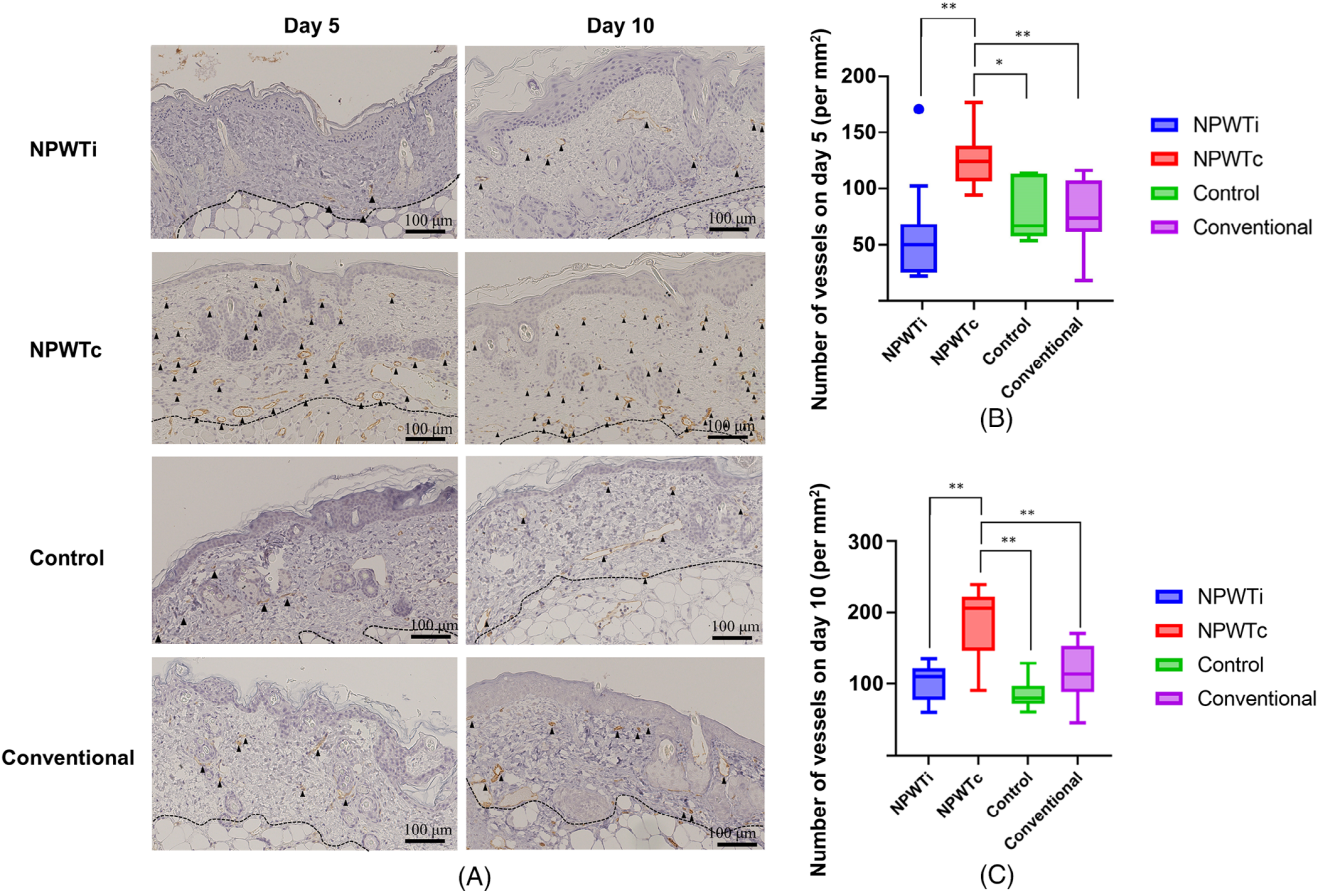
Number of capillaries in each group on days 5 and 10. (A) Representative images of immunohistochemical staining with CD31 antibody from each group (original magnification: ×200). Arrowheads indicate the capillaries. Broken lines show the boundary between the wound bed and skin graft. (B) Comparison of the number of capillaries on day 5. The number of capillaries in the dermis of the skin grafts on day 5 was significantly higher in the NPWTc group than in the other groups. (C) Comparison of the number of capillaries on day 10. On day 10, the number of capillaries in the dermis of the skin grafts was significantly higher in the NPWTc group than in the other groups. * *p* < 0.05. ** *p* < 0.01. NPWTc, Continuous negative‐pressure wound therapy; NPWTi, Intermittent negative‐pressure wound therapy. Scale bar = 100 µm.

The number of capillaries in the dermis of FTSGs on day 5 in the NPWTc group (126.4 ± 7.86) was significantly higher than in the other groups (NPWTi group: 62.3 ± 12.5, *p < *0.0002; conventional group: 77.6 ± 8.44, *p *= 0.0044; control group: 81.9 ± 9.23, *p *= 0.0213) (Figure 4). On day 10, the number of capillaries in the dermis of mice in the NPWTc group (187.9 ± 14.6) was significantly higher than in the other groups (NPWTi group: 102.7 ± 7.68, *p *< 0.0001; conventional group: 117.9 ± 13.6, *p = *0.0007; control group: 86.2 ± 7.39, *p *< 0.0001).

### 3.3 Levels of growth factors in the wound bed

**FIGURE 5:**
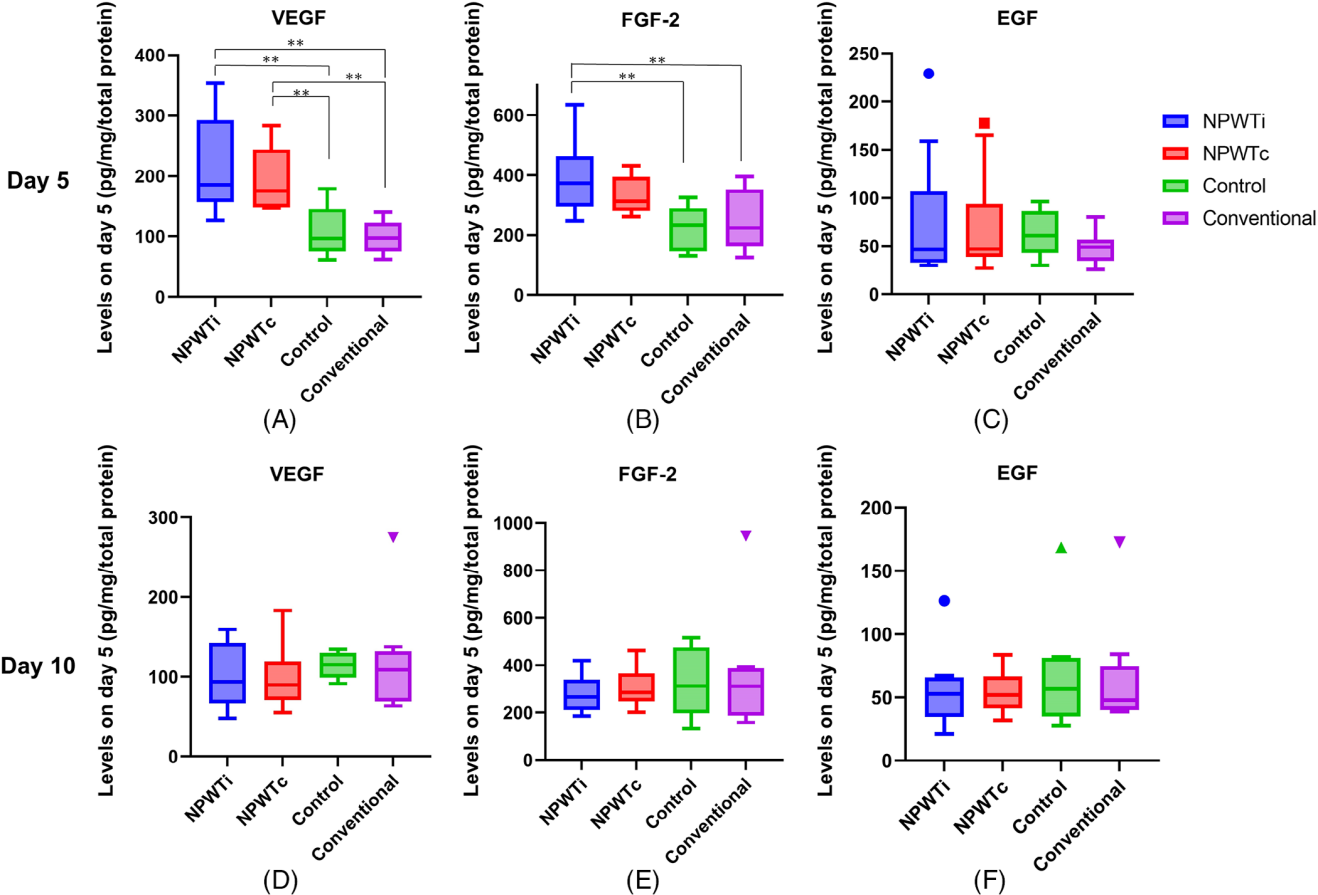
Comparison of VEGF, FGF, and EGF levels in mice after skin grafting. (A) VEGF levels on day 5. The VEGF levels in the wound bed on day 5 in the NPWTi and NPWTc groups were significantly higher than those in the control and conventional groups. (B) FGF‐2 levels on day 5. The FGF‐2 levels in the wound bed on day 5 in the NPWTi group were significantly higher than those in the control and conventional groups. (C) EGF levels on day 5. (D) VEGF levels on day 10. (E) FGF‐2 levels on day 10. (F) EGF levels on day 10. There were no significant differences in the levels of VEGF, FGF‐2, and EGF on day 10 among all groups. ** *p* < 0.01. EGF, Epidermal growth factor; FGF‐2, Basic fibroblast growth factor; NPWTc, Continuous negative‐pressure wound therapy; NPWTi, Intermittent negative‐pressure wound therapy; VEGF, Vascular endothelial growth factor.

VEGF levels in the wound bed on day 5 in the NPWTi (226.7 ± 24.3 pg/mg·total protein) and NPWTc (196.5 ± 16.0 pg/mg·total protein) groups were significantly higher than those in the control (110.0 ± 14.4 pg/mg·total protein, *p *= 0.0003, *p *= 0.0090, respectively) and conventional (101.7 ± 8.39 pg/mg·total protein, *p *< 0.0001, *p *= 0.0014, respectively) groups. FGF‐2 levels on day 5 in the NPWTi group (393.2 ± 35.9 pg/mg·total protein) were significantly higher than those in the control (225.2 ± 26.1 pg/mg·total protein, *p *= 0.0018) and conventional (248.2 ± 29.2 pg/mg·total protein, *p *= 0.0035) groups. In contrast, the FGF‐2 levels in the NPWTc group (328.6 ± 17.6 pg/mg·total protein) on day 5 were not significantly higher than those in the other groups. No significant differences in the levels of VEGF, FGF‐2, and EGF were noted on day 10 among all groups (Figure 5 and Table 2).

**TABLE 2:** Amount of each growth factor in mice after skin grafting.

	Amount of growth factor (pg/mg·total protein)							
	NPWTi		NPWTc		Control		Conventional	
	Day 5 (*n *= 11)	Day 10 (*n *= 10)	Day 5 (*n *= 11)	Day 10 (*n *= 10)	Day 5 (*n *= 8)	Day 10 (*n *= 8)	Day 5 (*n *= 11)	Day 10 (*n *= 9)
VEGF	226.7±24.3	101.7±12.3	196.5±15.9	97.9±12.3	110.0±14.4	114.4±5.87	101.7±8.39	114.9±22.0
FGF‐2	393.2±35.9	280.7±25.1	328.6±17.5	306.4±26.9	225.2±26.1	325.5±49.2	248.2±29.2	343.2±80.9
EGF	76.4±19.4	55.5±9.3	69.2±16.2	55.1±5.52	62.1±8.25	68.7±15.9	48.9±5.13	66.1±14.3

*Note*: The amount of each growth factor is expressed as mean ± SEM.

Abbreviations: EGF, Epidermal growth factor; FGF‐2, Basic fibroblast growth factor; NPWTc, Continuous negative‐pressure wound therapy; NPWTi, Intermittent negative‐pressure wound therapy; VEGF, Vascular endothelial growth factor.

## 4 DISCUSSION

In this study, we investigated the efficacy of NPWT in improving the survival rates of FTSGs in a mouse skin defect model. The efficacy of NPWT in wound healing has been observed in in vitro, in vivo, and clinical studies.^25^, ^27^, ^32^, ^33^ Although fixation of skin grafts using NPWT increased the survival rates of FTSG in several studies, almost all of these were clinical studies in which the biological mechanism underlying the efficacy of NPWT in enhancing skin graft viability was not investigated.^10^, ^11^, ^12^, ^13^, ^14^, ^15^, ^16^, ^17^, ^18^, ^19^, ^20^ While a few in vivo studies have reported the effectiveness of skin grafts with NPWT, these studies primarily used porcine models.^34^, ^35^ Thus, their effectiveness was limited to the evaluation of survival rates and vascularity in skin grafts. Several reports have indicated that NPWT promotes the secretion of growth factors from surrounding tissue through mechanical stress.^36^, ^37^, ^38^, ^39^, ^40^, ^41^ Moreover, previous literature has demonstrated improved skin graft survival with the administration of growth factors.^5^ However, there has been no direct examination of whether these two phenomena are related to the enhanced skin graft survival rate observed with NPWT. We aimed to elucidate the mechanism behind the efficacy of NPWT. On day 5, the survival rates of FTSGs fixed using NPWT were equivalent to the rates for those fixed using the conventional method, regardless of the mode of application of negative pressure (continuous or intermittent). However, on day 10, the survival rates of FTSGs fixed using the continuous mode of NPWT were significantly higher than those of the other groups. The number of capillaries in the dermis of the FTSGs was significantly higher in the NPWTc group than in the other groups on days 5 and 10. VEGF levels were significantly higher in both the NPWT groups than in the other groups on day 5.

Previous studies have shown that NPWT improves wound healing by increasing the discharge of various growth factors, cytokines, and chemokines from the surrounding tissue and cells, including macrophages, fibroblasts, and neutrophils.^36^, ^37^, ^38^, ^39^, ^40^, ^41^, ^42^ This effect is a consequence of the microdeformation of NPWT.^43^ The negative pressure in NPWT causes the movement of fluids via the cellular matrix, which results in the exertion of shear and deformation forces on the cells. The stimulation of cells disrupts integrin, and they release intracellular messengers, including growth factors and cytokine.^44^ Adherence of the polyurethane foam of NPWT and wound surface is important for achieving this effect. NPWT also increased the VEGF levels in the wound bed, even when the grafted skin was placed between NPWT and the wound bed, indicating microdeformation on the wound surface even through the grafted skin. The FGF‐2 levels were increased only in the NPWT group with intermittent application of pressure and not in the continuous mode. These results are consistent with those of previous studies,^22^, ^45^ which indicated that the intermittent mode of NPWT was more effective in creating good granulation than the continuous mode. However, whether NPWT augments the FGF‐2 levels in the wound tissue remains controversial. In a rodent model, the expression of FGF‐2 exhibited a biphasic response, with a significant increase on day 5, followed by a significant decrease on day 7^41^ whereas NPWT did not affect FGF‐2 expression in a clinical study.^37^

Postoperative administration of various growth factors improves the survival rate of skin grafts. For example, VEGF, FGF‐2, and EGF improve the survival rate and quality of skin grafts, including color matching, pigmentation, and contracture.^5^, ^46^, ^47^, ^48^ VEGF and FGF‐2 are particularly potent angiogenic agents.^49^, ^50^ Therefore, their administration promoted the revascularization of skin grafts in previous studies. Recent reports have shown that the administration of agents containing many growth factors and cytokines is more effective in improving skin graft survival than the administration of a single growth factor. Adipose‐derived stem cells (ASC), which contain many growth factors including VEGF, FGF‐2, and insulin growth factor‐1, improve isogenic skin graft survival and prolong allogeneic skin graft survival through immunomodulatory activity.^51^, ^52^, ^53^ Platelet‐rich plasma (PRP) promotes revascularization of skin grafts and shortens the period of tie‐over fixation in rats.^54^ NPWT, ASC, and PRP induce the release of various growth factors and cytokines from surrounding tissues. This explains why the number of vessels in the skin graft was greater in the NPWTc group than in the other groups.

Fixing the graft on the wound bed is important for graft survival. Even when a skin graft is placed on a well‐vascularized wound bed, there is initially no vascular connection between the graft and the wound bed. Initially, the graft is nourished only by the plasma exudation from capillaries into the wound bed. However, over time, graft revascularization occurs through direct anastomosis between the graft and wound bed. Fixation of the skin graft, such as tie‐over, is required to complete anastomosis. The time required for the completion of anastomosis depends on the degree of vascularization of the wound bed. NPWT creates suitable wound beds for skin grafts by increasing the levels of growth factors and cytokines. This effect is more potent in the intermittent mode of NPWT than in the continuous mode.^22^, ^41^ In our study, regardless of the NPWTi‐induced increase in VEGF and FGF‐2 levels in the wound bed, the survival rates of FTSGs on day 10 in the NPWTi group were as high as those in the control and conventional groups. The black polyurethane foam used to fix the graft repeatedly contracted and expanded in the intermittent mode of NPWT, which could have shifted the graft slightly and prevented revascularization.

The appropriate covering time for NPWT for skin graft survival remains unclear. In cohort studies and randomized controlled trials, it was reported to be 4−7 days. Although the survival rates on day 5 were not significantly different among the NPWTc, NPWTi, and conventional groups in the present study, those on day 10 were significantly higher in the NPWTc group than in the NPWTi and conventional groups. Moreover, the survival rates on days 5 and 10 decreased in the NPWTi and conventional groups but not in the NPWTc group. The results indicate that fixation of FTSGs using the continuous mode of NPWT shortened the time required for skin graft survival, contributing to the shortening of the duration of immobilization, accelerating the start of rehabilitation, and decreasing hospitalization.

This study had two limitations. Specimens were excluded if the survival rate of the skin grafts was less than 50%. This was because the living tissue was too small to examine histopathological staining and measure growth factors. Thus, we could not obtain immunohistopathological and growth factor data for necrotizing tissue. Second, the power‐on time of the vacuum system was short in compliance with the instructions of the Animal Research Committee of our institution. However, despite the short treatment duration, our results indicate that skin graft fixation using NPWT was effective.

## 5 CONCLUSION

We investigated the efficacy of NPWT in improving the survival rate of FTSGs in a mouse skin defect model. Our results indicate that continuous NPWT effectively increases the survival rate of FTSGs and shortens the time required for skin graft survival.

## CONFLICT OF INTEREST STATEMENT

The authors declare no conflict of interest.

## ETHICS STATEMENT

All animal experiments in this study were performed in accordance with the ARRIVE guidelines, and the experimental protocol was approved by the Defense Medical College Animal Experiment Ethics Committee (approval number: 190057). All procedures were performed in accordance with the relevant guidelines and regulations of the National Defense Medical College.

## Data Availability

The datasets generated during and/or analyzed during the current study are available from the corresponding author upon reasonable request.

## References

[1] Chung KC . Grabb and Smith's Plastic Surgery. 9th ed. Wolters Kluwer/Lippincott Williams & Wilkins Health; 2024.

[2] Hjort A , Gottrup F . Cost of wound treatment to increase significantly in Denmark over the next decade. J Wound Care. 2010;19(5):173‐184. 20505590 10.12968/jowc.2010.19.5.48046

[3] Hill TG . Enhancing the survival of full‐thickness grafts. J Dermatol Surg Oncol. 1984;10(8):639‐642. 6379012 10.1111/j.1524-4725.1984.tb01269.x

[4] Khan RJ , Fick D , Yao F , et al. A comparison of three methods of wound closure following arthroplasty: a prospective, randomised, controlled trial. J Bone Joint Surg Br. 2006;88(2):238‐242. 16434531 10.1302/0301-620X.88B2.16923

[5] Zhang F , Lineaweaver W . Acute and sustained effects of vascular endothelial growth factor on survival of flaps and skin grafts. Ann Plast Surg. 2011;66(5):581‐582. 21451385 10.1097/SAP.0b013e3182057376

[6] Currie LJ , Sharpe JR , Martin R . The use of fibrin glue in skin grafts and tissue‐engineered skin replacements: a review. Plast Reconstr Surg. 2001;108(6):1713‐1726. 11711954 10.1097/00006534-200111000-00045

[7] Muneuchi G , Suzuki S , Moriue T , Igawa HH . Combined treatment using artificial dermis and basic fibroblast growth factor (bFGF) for intractable fingertip ulcers caused by atypical burn injuries. Burns. 2005;31(4):514‐517. 15896519 10.1016/j.burns.2004.11.016

[8] Saber AA , Yahya KZ , Rao A , et al. A new approach in the management of chronic nonhealing leg ulcers. J Invest Surg. 2005;18(6):321‐323. 16319053 10.1080/08941930500328821

[9] Zografou A , Papadopoulos O , Tsigris C , et al. Autologous transplantation of adipose‐derived stem cells enhances skin graft survival and wound healing in diabetic rats. Ann Plast Surg. 2013;71(2):225‐232. 23636118 10.1097/SAP.0b013e31826af01a

[10] Bach CA , Guilleré L , Yildiz S , Wagner I , Darmon S , Chabolle F . Comparison of negative pressure wound therapy and conventional dressing methods for fibula free flap donor site management in patients with head and neck cancer. Head Neck. 2016;38(5):696‐699. 25522136 10.1002/hed.23952

[11] Blume PA , Key JJ , Thakor P , Thakor S , Sumpio B . Retrospective evaluation of clinical outcomes in subjects with split‐thickness skin graft: comparing V.A.C.^®^ therapy and conventional therapy in foot and ankle reconstructive surgeries. Int Wound J. 2010;7(6):480‐487. 20825510 10.1111/j.1742-481X.2010.00728.x PMC7951281

[12] Ho MW , Rogers SN , Brown JS , Bekiroglu F , Shaw RJ . Prospective evaluation of a negative pressure dressing system in the management of the fibula free flap donor site: a comparative analysis. JAMA Otolaryngol Head Neck Surg. 2013;139(10):1048‐1053. 24008650 10.1001/jamaoto.2013.4544

[13] Hsiao SF , Ma H , Wang YH , Wang TH . Occlusive drainage system for split‐thickness skin graft: a prospective randomized controlled trial. Burns. 2017;43(2):379‐387. 28341261 10.1016/j.burns.2016.08.025

[14] Kim EK , Hong JP . Efficacy of negative pressure therapy to enhance take of 1‐stage allodermis and a split‐thickness graft. Ann Plast Surg. 2007;58(5):536‐540. 17452839 10.1097/01.sap.0000245121.32831.47

[15] Lee KT , Pyon JK , Lim SY , Mun GH , Oh KS , Bang SI . Negative‐pressure wound dressings to secure split‐thickness skin grafts in the perineum. Int Wound J. 2014;11(2):223‐227. 22958590 10.1111/j.1742-481X.2012.01078.x PMC7951014

[16] Llanos S , Danilla S , Barraza C , et al. Effectiveness of negative pressure closure in the integration of split thickness skin grafts: a randomized, double‐masked, controlled trial. Ann Surg. 2006;244(5):700‐705. 17060762 10.1097/01.sla.0000217745.56657.e5 PMC1856589

[17] Moisidis E , Heath T , Boorer C , Ho K , Deva AK . A prospective, blinded, randomized, controlled clinical trial of topical negative pressure use in skin grafting. Plast Reconstr Surg. 2004;114(4):917‐922. 15468399 10.1097/01.prs.0000133168.57199.e1

[18] Scherer LA , Shiver S , Chang M , Meredith JW , Owings JT . The vacuum assisted closure device: a method of securing skin grafts and improving graft survival. Arch Surg. 2002;137(8):933–934. 10.1001/archsurg.137.8.930 12146992

[19] Wu CC , Chew KY , Chen CC , Kuo YR . Antimicrobial‐impregnated dressing combined with negative‐pressure wound therapy increases split‐thickness skin graft engraftment: a simple effective technique. Adv Skin Wound Care. 2015;28(1):21‐27. 25502972 10.1097/01.ASW.0000459038.81701.fb

[20] Zhang F , Lv KY , Qiu XC , et al. Using negative pressure wound therapy on microskin autograft wounds. J Surg Res. 2015;195(1):344‐350. 25586332 10.1016/j.jss.2014.12.025

[21] Kamolz LP , Lumenta DB . Topical negative pressure therapy for skin graft fixation in hand and feet defects: a method for quick and easy dressing application—the “sterile glove technique”. Burns. 2013;39(4):814‐815. 23092700 10.1016/j.burns.2012.09.019

[22] Morykwas MJ , Argenta LC , Shelton‐Brown EI , McGuirt W . Vacuum‐assisted closure: a new method for wound control and treatment: animal studies and basic foundation. Ann Plast Surg. 1997;38(6):553‐562. 9188970 10.1097/00000637-199706000-00001

[23] Torbrand C , Ugander M , Engblom H , Arheden H , Ingemansson R , Malmsjö M . Wound contraction and macro‐deformation during negative pressure therapy of sternotomy wounds. J Cardiothorac Surg. 2010;5:75. 20920290 10.1186/1749-8090-5-75 PMC2958889

[24] Zeybek B , Li S , Silberschmidt VV , Liu Y . Wound contraction under negative pressure therapy measured with digital image correlation and finite‐element analysis in tissue phantoms and wound models. Med Eng Phys. 2021;98:104‐114. 34848029 10.1016/j.medengphy.2021.11.003

[25] Hsu CC , Tsai WC , Chen CP , Lu YM , Wang JS . Effects of negative pressures on epithelial tight junctions and migration in wound healing. Am J Physiol Cell Physiol. 2010;299(2):C528‐C534. 20445172 10.1152/ajpcell.00504.2009

[26] Hsu CC , Chow SE , Chen CP , et al. Negative pressure accelerated monolayer keratinocyte healing involves Cdc42 mediated cell podia formation. J Dermatol Sci. 2013;70(3):196‐203. 23622765 10.1016/j.jdermsci.2013.03.007

[27] Zhu J , Yu A , Qi B , Li Z , Hu X . Effects of negative pressure wound therapy on mesenchymal stem cells proliferation and osteogenic differentiation in a fibrin matrix. PLoS One. 2014;9(9):e107339. 25216182 10.1371/journal.pone.0107339 PMC4162584

[28] Ngo QD , Vickery K , Deva AK . The effect of topical negative pressure on wound biofilms using an in vitro wound model. Wound Repair Regen. 2012;20(1):83‐90. 22126340 10.1111/j.1524-475X.2011.00747.x

[29] Janssen AH , Mommers EH , Notter J , de Vries Reilingh TS , Wegdam JA . Negative pressure wound therapy versus standard wound care on quality of life: a systematic review. J Wound Care. 2016;25(3):154‐159. 26947696 10.12968/jowc.2016.25.3.154

[30] Liu X , Zhang H , Cen S , Huang F . Negative pressure wound therapy versus conventional wound dressings in treatment of open fractures: a systematic review and meta‐analysis. Int J Surg. 2018;53:72‐79. 29555530 10.1016/j.ijsu.2018.02.064

[31] Cheng CH , Lee CF , Fryer M , et al. Murine full‐thickness skin transplantation. JoVE. 2017(119):e55105. 10.3791/55105 PMC5408726 28117792

[32] Apelqvist J , Willy C , Fagerdahl AM , et al. EWMA document: negative pressure wound therapy. J Wound Care. 2017;26(Sup3):S1‐S154. 10.12968/jowc.2017.26.Sup3.S1 28345371

[33] Wang HP , Hu XL , Zhou HQ , Chen XS , Wang MX . The safety and effectiveness of a self‐made negative pressure suction device in the treatment of chronic wounds. Skin Res Technol. 2023;29(12):e13415. 38062667 10.1111/srt.13415 PMC10704041

[34] Carney BC , Moffatt LT , Travis TE , et al. A pilot study of negative pressure therapy with autologous skin cell suspensions in a porcine model. J Surg Res. 2021;267:182‐196. 34153561 10.1016/j.jss.2021.05.010

[35] Ward C , Ciraulo D , Coulter M , Desjardins S , Liaw L , Peterson S . Does treatment of split‐thickness skin grafts with negative‐pressure wound therapy improve tissue markers of wound healing in a porcine experimental model? J Trauma Acute Care Surg. 2012;73(2):447‐451. 22846954 10.1097/TA.0b013e31825aa9ea PMC3698483

[36] Lu F , Ogawa R , Nguyen DT , et al. Microdeformation of three‐dimensional cultured fibroblasts induces gene expression and morphological changes. Ann Plast Surg. 2011;66(3):296‐300. 21233699 10.1097/SAP.0b013e3181ea1e9b

[37] Labler L , Rancan M , Mica L , Härter L , Mihic‐Probst D , Keel M . Vacuum‐assisted closure therapy increases local interleukin‐8 and vascular endothelial growth factor levels in traumatic wounds. J Trauma. 2009;66(3):749‐757. 19276749 10.1097/TA.0b013e318171971a

[38] Stechmiller JK , Kilpadi DV , Childress B , Schultz GS . Effect of vacuum‐assisted closure therapy on the expression of cytokines and proteases in wound fluid of adults with pressure ulcers. Wound Repair Regen. 2006;14(3):371‐373. 16808818 10.1111/j.1743-6109.2006.00134.x

[39] Eisenhardt SU , Schmidt Y , Thiele JR , et al. Negative pressure wound therapy reduces the ischaemia/reperfusion‐associated inflammatory response in free muscle flaps. J Plast Reconstr Aesthet Surg. 2012;65(5):640‐649. 22137686 10.1016/j.bjps.2011.11.037

[40] Glass GE , Murphy GF , Esmaeili A , Lai LM , Nanchahal J . Systematic review of molecular mechanism of action of negative‐pressure wound therapy. Br J Surg. 2014;101(13):1627‐1636. 25294112 10.1002/bjs.9636

[41] Jacobs S , Simhaee DA , Marsano A , Fomovsky GM , Niedt G , Wu JK . Efficacy and mechanisms of vacuum‐assisted closure (VAC) therapy in promoting wound healing: a rodent model. J Plast Reconstr Aesthet Surg. 2009;62(10):1331‐1338. 18617451 10.1016/j.bjps.2008.03.024

[42] Yamashiro T , Kushibiki T , Mayumi Y , Tsuchiya M , Ishihara M , Azuma R . Novel cell culture system for monitoring cells during continuous and variable negative‐pressure wound therapy. Skin Res Technol. 2023;29(1):e13262. 36704879 10.1111/srt.13262 PMC9838773

[43] Normandin S , Safran T , Winocour S , et al. Negative pressure wound therapy: mechanism of action and clinical applications. Semin Plast Surg. 2021;35(3):164‐170. 34526864 10.1055/s-0041-1731792 PMC8432996

[44] Huang S , Chen CS , Ingber DE . Control of cyclin D1, p27(Kip1), and cell cycle progression in human capillary endothelial cells by cell shape and cytoskeletal tension. Mol Biol Cell. 1998;9(11):3179‐3193. 9802905 10.1091/mbc.9.11.3179 PMC25607

[45] Malmsjö M , Gustafsson L , Lindstedt S , Gesslein B , Ingemansson R . The effects of variable, intermittent, and continuous negative pressure wound therapy, using foam or gauze, on wound contraction, granulation tissue formation, and ingrowth into the wound filler. Eplasty. 2012;12:e5. 22292101 PMC3266212

[46] Zhang F , Oswald TM , Lin L , Wang S , Lin S , Lineaweaver WC . Improvement of full‐thickness skin graft survival by application of vascular endothelial growth factor in rats. Ann Plast Surg. 2008;60(5):589‐593. 18434837 10.1097/SAP.0b013e31816d78fe

[47] Akita S , Akino K , Imaizumi T , Hirano A . A basic fibroblast growth factor improved the quality of skin grafting in burn patients. Burns. 2005;31(7):855‐858. 16199295 10.1016/j.burns.2005.04.008

[48] Cooper ML , Hansbrough JF , Foreman TJ , Sakabu SA , Laxer JA . The effects of epidermal growth factor and basic fibroblast growth factor on epithelialization of meshed skin graft interstices. Prog Clin Biol Res. 1991;365:429‐442. 1862148

[49] Hoeben A , Landuyt B , Highley MS , Wildiers H , Van Oosterom AT , De Bruijn EA . Vascular endothelial growth factor and angiogenesis. Pharmacol Rev. 2004;56(4):549‐580. 15602010 10.1124/pr.56.4.3

[50] Rusnati M , Presta M . Interaction of angiogenic basic fibroblast growth factor with endothelial cell heparan sulfate proteoglycans. Biological implications in neovascularization. Int J Clin Lab Res. 1996;26(1):15‐23. 8739851 10.1007/BF02644769

[51] Hu JL , Kim BJ , Yu NH , Kwon ST . Impact of Injection frequency of adipose‐derived stem cells on allogeneic skin graft survival outcomes in mice. Cell Transplant. 2021;30:096368972110419. 10.1177/09636897211041966 PMC8743972 34538121

[52] Sotoodehnejadnematalahi F , Moghadasali R , Hajinasrollah M , et al. Immunomodulatory activity of human bone marrow and adipose‐derived mesenchymal stem cells prolongs allogenic skin graft survival in nonhuman primates. Cell J. 2021;23(1):1‐13. 33650815 10.22074/cellj.2021.6895 PMC7944119

[53] Vidor SB , Terraciano PB , Valente FS , et al. Adipose‐derived stem cells improve full‐thickness skin grafts in a rat model. Res Vet Sci. 2018;118:336‐344. 29621642 10.1016/j.rvsc.2018.03.014

[54] Takabayashi Y , Ishihara M , Kuwabara M , et al. Improved survival of full‐thickness skin graft with low‐molecular weight heparin‐protamine micro/nanoparticles including platelet‐rich plasma. Ann Plast Surg. 2017;78(5):562‐568. 28272145 10.1097/SAP.0000000000001051

